# Quantification of the misidentification of *Plasmodium knowlesi* as *Plasmodium malariae* by microscopy: an analysis of 1569 *P. knowlesi* cases

**DOI:** 10.1186/s12936-021-03714-1

**Published:** 2021-04-09

**Authors:** Aongart Mahittikorn, Frederick Ramirez Masangkay, Kwuntida Uthaisar Kotepui, Giovanni De Jesus Milanez, Manas Kotepui

**Affiliations:** 1 Department of Protozoology, Faculty of Tropical Medicine, Mahidol University, Bangkok, Thailand; 2 Department of Medical Technology, Institute of Arts and Sciences, Far Eastern University-Manila, Manila, Philippines; 3 Medical Technology, School of Allied Health Sciences, Walailak University, Tha Sala, Nakhon Si Thammarat, Thailand

**Keywords:** *P. knowlesi*, *P. malariae*, *Plasmodium*, Malaria, Microscopy, Monkey, Blood

## Abstract

**Background:**

*Plasmodium knowlesi* is recognized as the fifth *Plasmodium* species causing malaria in humans. It is morphologically similar to the human malaria parasite *Plasmodium malariae*, so molecular detection should be used to clearly discriminate between these *Plasmodium* species. This study aimed to quantify the rate at which *P. knowlesi* is misidentified as *P. malariae* by microscopy in endemic and non-endemic areas.

**Methods:**

The protocol of this systematic review was registered in the PROSPERO International Prospective Register of Systematic Reviews (ID = CRD42020204770). Studies reporting the misidentification of *P. knowlesi* as *P. malariae* by microscopy and confirmation of this by molecular methods in MEDLINE, Web of Science and Scopus were reviewed. The risk of bias in the included studies was assessed using the Quality Assessment of Diagnostic Accuracy Studies (QUADAS). The pooled prevalence and 95% confidence interval (CI) of the misidentification of *P. knowlesi* as *P. malariae* by microscopy were estimated using a random effects model. Subgroup analysis of the study sites was performed to demonstrate any differences in the misidentification rates in different areas. Heterogeneity across the included studies was assessed and quantified using Cochran’s Q and I^2^ statistics, respectively. Publication bias in the included studies was assessed using the funnel plot, Egger’s test and contour-enhanced funnel plot.

**Results:**

Among 375 reviewed studies, 11 studies with a total of 1569 confirmed *P. knowlesi* cases in humans were included. Overall, the pooled prevalence of the misidentification of *P. knowlesi* as *P. malariae* by microscopy was estimated at 57% (95% CI 37–77%, I^2^: 99.3%). Subgroup analysis demonstrated the highest rate of misidentification in Sawarak, Malaysia (87%, 95% CI 83–90%, I^2^: 95%), followed by Sabah, Malaysia (85%, 95% CI 79–92%, I^2^: 85.1%), Indonesia (16%, 95% CI 6–38%), and then Thailand (4%, 95% CI 2–9%, I^2^: 95%).

**Conclusion:**

Although the World Health Organization (WHO) recommends that all *P. malariae*-positive diagnoses made by microscopy in *P. knowlesi* endemic areas be reported as *P. malariae*/*P. knowlesi* malaria, the possibility of microscopists misidentifying *P. knowlesi* as *P. malariae* is a diagnostic challenge. The use of molecular techniques in cases with *malariae*-like *Plasmodium* with high parasite density as determined by microscopy could help identify human *P. knowlesi* cases in non-endemic countries.

**Supplementary Information:**

The online version contains supplementary material available at 10.1186/s12936-021-03714-1.

## Background

*Plasmodium knowlesi* was first recognized as a cause of simian malaria in long-tailed or pig-tailed macaques [1]. It was also recognized as a cause of human malaria in 1965 [2]. The large focus on *P. knowlesi* as a cause of human malaria was reported in Sarawak, Malaysia, in 2004 [3]. Since then, *P. knowlesi* malaria in humans has been reported throughout Malaysia [4–22] and other Southeast Asian countries including Thailand [23–26], Indonesia [27–30], Singapore [31, 32], Brunei [33], Cambodia [34, 35], Laos [36, 37], Myanmar [38], the Philippines [39], and Vietnam [40]. Moreover, *P. knowlesi* malaria has also been reported in travellers returning from endemic countries [41–54].

Although most *P. knowlesi* infections are asymptomatic, approximately 19% of infected patients develop severe infections, including acute kidney injury (AKI) (45.6%), jaundice (42%), and hyperparasitaemia (32.5%), as the common clinical manifestations [55]. Deaths from *P. knowlesi* infection have been linked to delayed parenteral treatment [56]. In the endemic country of Malaysia, early intravenous artesunate treatment is now recommended for all severe malaria cases to prevent mortality, resulting in a lower death rate during 2010–2014 [8]. The risk factors associated with *P. knowlesi* infection include older age, male sex, plantation work, sleeping outside, and travelling in areas where monkeys live [21, 55, 57]. A recent study also suggested that the transmission of *P. knowlesi* malaria between humans might occur with mosquitoes as vectors, given the presence of family clustering [14].

The identification or detection of malaria parasites relies on the results of analysis based on microscopy, the standard for malaria diagnosis. However, the use of microscopy to diagnose *P. knowlesi* malaria is inaccurate since the morphological features of the early trophozoites of *P. knowlesi* resemble those of *Plasmodium falciparum*, and the growing trophozoites are similar to the band-form trophozoites of *Plasmodium malariae* [1, 58]. In this study, the rate at which *P. knowlesi* is misidentified as *P. malariae* by microscopy was estimated and quantified to clarify the inherent disadvantage of solely utilizing microscopy to identify *P. knowlesi* infection in endemic and non-endemic areas.

## Methods

This study followed the Preferred Reporting Items for Systematic Reviews and Meta-Analyses (PRISMA) (Additional file 1: Checklist S1) [59]. The protocol was registered in the PROSPERO International Prospective Register of Systematic Reviews (ID = CRD42020204770).

### Search strategy

Searches of potentially relevant articles published from January 1, 1993, to August 17, 2020 were performed in MEDLINE, Scopus and Web of Science. The search terms used were (*Plasmodium* OR malaria) AND *knowlesi* AND (microscopy OR microscopic OR blood film OR “blood film” OR “thick film” OR “thin film”) AND (PCR OR “polymerase chain reaction”). The searches aimed to find original articles in any language and ended on August 17, 2020.

### Eligibility criteria

Original research articles were eligible to be included in the present study if they were on retrospective or prospective cross-sectional studies and reported: (1) the misidentification of *P. knowlesi* as *P. malariae* as identified by microscopy and (2) the confirmation of *P. knowlesi* cases by molecular methods. Studies/papers were excluded for the following reasons: absence of *P. malariae* or *P. knowlesi* as determined by microscopy, absence of *P. knowlesi* as determined by PCR, microscopic findings of *P. malariae*/*P. knowlesi*, *P. knowlesi* in macaques, submicroscopic *P. knowlesi*, unextractable data, case–control studies, case reports or case series, clinical trials, conference abstracts, experimental research, guidelines, letters to the editor, test performances, review articles, systematic reviews and use of the same participants or data set as in another study.

### Study selection and data extraction

The selection of the included studies according to the eligibility criteria was performed by two of the authors (MK and AM). Any discrepancies between these two authors were resolved by discussion in order to reach a consensus. For each study that was included in the analysis, the following information was extracted: name of the first author, year of publication, study area (years of the survey), study design, age range (years) of the participants, sex (male, %) of the participants, PCR detection for *Plasmodium* spp., target genes, number of *P. malariae* and *P. knowlesi* identified by microscopy (including mixed infections), number of *P. malariae* and *P. knowlesi* identified by PCR (including mixed infections), and number of species discordances. Raw data from each study were stored in a standardized datasheet before data synthesis. Data selection and extraction were managed using Endnote Software X7 (Clarivate Analytics, Philadelphia, USA).

### Quality of the included studies

The risk of bias for each study was assessed using the Quality Assessment of Diagnostic Accuracy Studies (QUADAS) [60]. This tool comprises four domains: patient selection, index test, reference standard, and flow and timing [60].

### Statistical analysis

Data from the included studies were analysed using the STATA Statistical Software Version 15.0 (StataCorp LLC, Texas, USA). The number of cases of *Plasmodium* species discordance (*P. knowlesi* as *P. malariae*) as identified by microscopy and the number of *P. knowlesi* cases identified by PCR were used to analyse the pooled prevalence of the misidentification of *P. knowlesi* as *P. malariae*. The pooled prevalence of discordance of the misidentification of *P. knowlesi* as *P. malariae* was estimated by a random effects model using the numerator in the prevalence calculation as the number of discordances, and the denominator as the number of PCR-positive malaria cases. The pooled prevalence and 95% confidence interval (CI) of the misidentification were estimated using a random effects model. Subgroup analysis of the study sites was performed to demonstrate any differences in the pooled prevalence in both endemic and non-endemic countries. The existence and level of heterogeneity across the included studies were assessed using Cochrane Q and I^2^ statistics, respectively. Publication bias was assessed using funnel plot asymmetry and Egger’s test for asymmetry.

## Results

### Search results

**Fig. 1:**
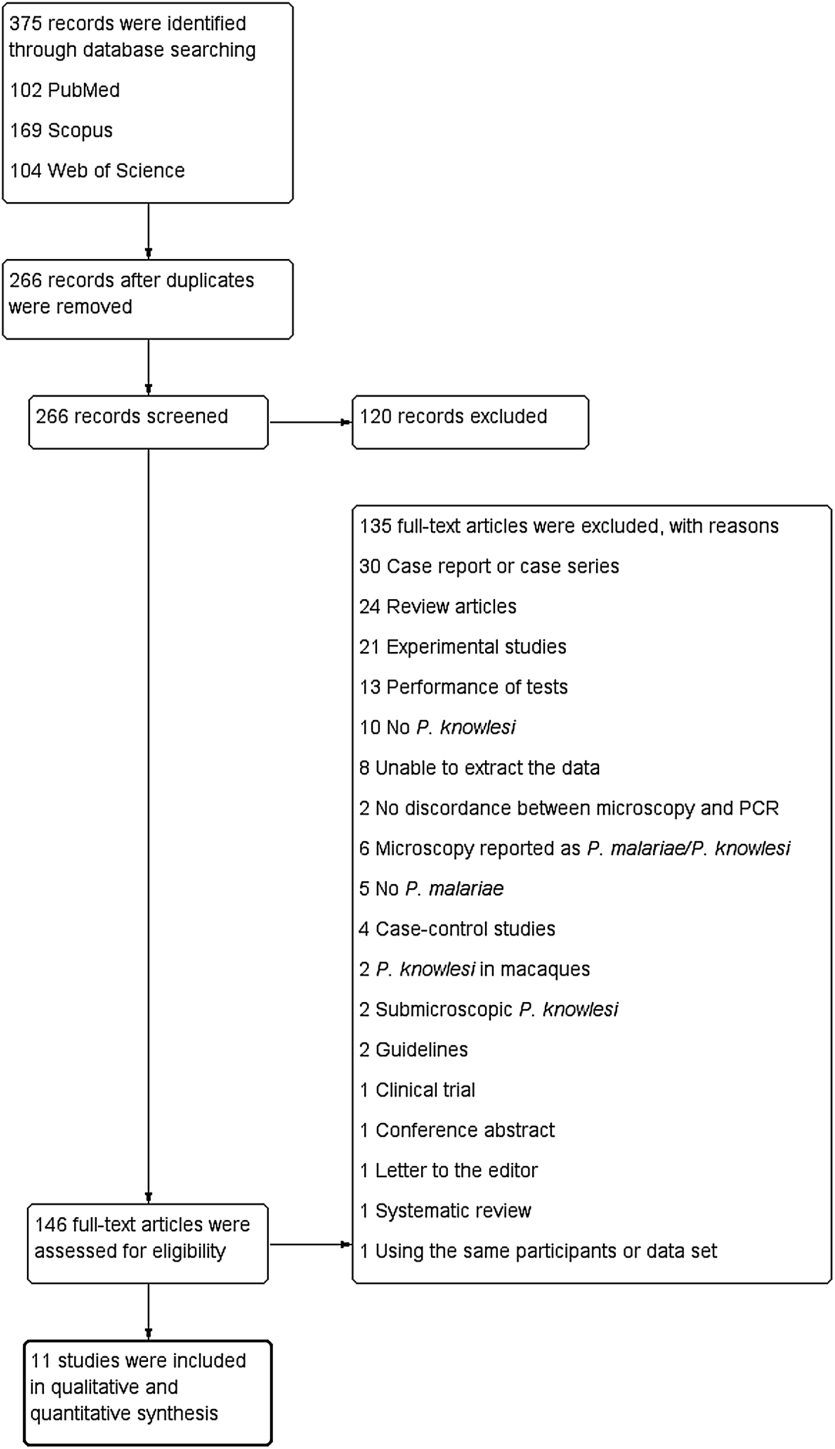
Flowchart for the study selection. Flowchart demonstrates the selection of potentially relevant studies for the present study

A total of 375 potentially relevant studies were identified from the searched databases. Among these, 109 were duplicates and removed. The papers on the remaining 266 studies were subjected to title and abstract screening. After this step, 146 papers were examined for their full text. Among these, 135 were excluded for the following reasons: no discordance between microscopy and PCR (n = 2), no *P. malariae* (n = 5), no *P. knowlesi* (n = 10), unextractable data (n = 8), case–control studies (n = 4), case reports or case series (n = 30), clinical trial (n = 1), conference abstract (n = 1), experimental studies (n = 21), guidelines (n = 2), letter to the editor (n = 1), microscopy findings reported as *P. malariae/P. knowlesi* (n = 6), *P. knowlesi* in macaques (n = 2), test performances (n = 13), review articles (n = 24), submicroscopic *P. knowlesi* (n = 2), systematic review (n = 1), and use of the same participants or data set (n = 1) (Fig. 1). Finally, a total of 11 studies [3, 13–19, 23, 24, 27] met the inclusion criteria and were included in the qualitative and quantitative synthesis.

### Characteristics of the included studies

The characteristics of the included studies are presented in Table 1. The largest proportion of the included studies (5/11, 45.5%) were conducted in Sabah [14, 15, 17–19], while two (18.2%) were in Sawarak [3, 19], two (18.2%) in Thailand [23, 24], one (9.1%) in Malaysia (nine states) [16], and one (9.1%) in Aceh, Indonesia [27]. Most of the included studies (6/11, 54.5%) were retrospective in nature, while five (5/11, 45.5%) were prospective. Seven studies (45.5%) [3, 14–17, 19, 23] reported information on the age and sex of patients infected with *P. knowlesi*. All of these seven studies reported mean/median age in the range of 11–91 years, and the proportion of males was between 70 and 77.9%. Seven studies [3, 13–16, 19, 27] enrolled malaria positive samples for the analysis, while four studies [17, 18, 23, 24] enrolled patients suspected of having malaria. In most of the studies [3, 13–17, 19, 23, 24, 27], a test of nested PCR amplifying 18S rRNA was performed to identify *P. knowlesi*, with the exception of the study by Goh et al. [18], in which a Hexaplex PCR test was performed. Based on the 11 included studies, 1894 malaria cases were identified by microscopy, while 7953 malaria cases were identified by PCR. Microscopy identified 1425 *P. malariae* cases, while PCR identified 45. Also, microscopy identified 182 *P. knowlesi* cases, while PCR identified 1569.

**Table 1:** Characteristics of the included studies

No.	Authors, year	Study area (years of the survey)	Study design	Age range (years)	Sex (male, %)	Participants	PCR for *Plasmodium* spp.	Target gene	Microscopy (including mixed infection)			PCR (including mixed infection)			No. of discordances
									No. of malaria	No. of *P. malariae*	No. of *P. knowlesi*	No. of malaria	No. of *P. malariae*	No. of *P. knowlesi*	
1	Anderios et al., 2008	Sabah, Malaysia	Retrospective cross-sectional study	NS	NS	31 *P. malariae*-positive by microscopy	Nested PCR	18S rRNA	31	31	0	31	0	25	25
2	Barber et al., 2012	Sabah, Malaysia (2009–2011)	Retrospective cross-sectional study	*P. knowlesi*: 33 years, IQR 20–50 years	*P. knowlesi*: 73%	485 malaria positive by microscopy	Nested PCR	18S rRNA	485	445	0	435	4	379	339
3	Coutrier et al., 2018	Aceh, Indonesia (2014–2015)	Prospective study	NS	NS	41 malaria positive by microscopy	Nested PCR	18S rRNA	41	3	0	41	0	19	3
4	Cox-Singh et al., 2008	Sarawak, Malaysia (2001–2006)	Retrospective cross-sectional study	Mean 36.9, 0.2–91 years	75.8%	960 malaria positive by microscopy	Nested PCR	18S rRNA	960	312	0	960	4	266	228
5	Goh et al., 2013	Sabah, Malaysia (2008–2011)	Prospective study	NS	NS	189 patients suspected of malaria	Hexaplex PCR	18S rRNA	189	49	0	178	2	42	35
6	Jongwutiwes et al., 2011	Thailand (2008–2009)	Retrospective and prospective study	Mean 27.4, 1–87 years	78%	3770 patients suspected of malaria	Nested PCR	18S rRNA	3300	2	0	3446	8	33	1
7	Joveen-Neoh et al., 2011	Sabah, Malaysia (2010)	Prospective study	11–20 years	74.5%	243 patients suspected of malaria	Nested PCR	18S rRNA	83	43	0	107	0	65	43
8	Naing et al., 2011	Sabah, Malaysia (2009)	Retrospective study	33 ± 18 years	73.8%	445 samples referred for PCR analysis	Nested PCR	NS	445	318	NS	343	2	343	316
9	Putaporntip et al., 2009	Thailand (2006–2007)	Prospective study	Malaria positive: mean 25.54 (1–81)	Malaria positive: 2.25:1	1874 patients suspected of malaria	Nested PCR	18S rRNA	1695	3	0	1751	24	10	1
10	Singh et al., 2004	Sarawak, Malaysia (2000–2002)	Prospective study	NS	NS	208 malaria positive by microscopy	Nested PCR	18S rRNA	208	141	0	208	0	120	106
11	Yusof et al., 2014	Malaysia (2012–2013)	Retrospective cross-sectional study	Mean 33.8	77.9%	457 malaria positive by microscopy	Nested PCR	18S rRNA	457	82	182	453	1	267	73

*NS* not specified

### Quality of the included studies

**Fig. 2:**
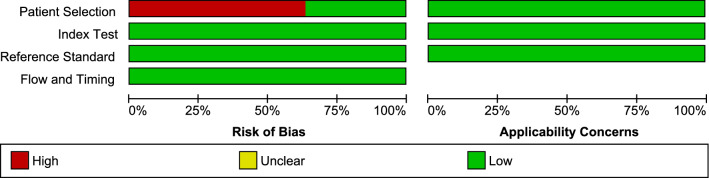
Methodological quality of the included studies. The quality of the included studies was assessed using QUADAS. Red indicates high bias while green indicates low bias

The risk of bias in each study was assessed using QUADAS. The results of the quality assessment are presented in Fig. 2 and Additional file 2. Seven studies (7/11, 63.6%) [3, 13–16, 19, 27] introduced bias in the selection of malaria positive samples for analysis.

### Prevalence of the misidentification of *P. knowlesi* as *P. malariae* by microscopy

**Fig. 3:**
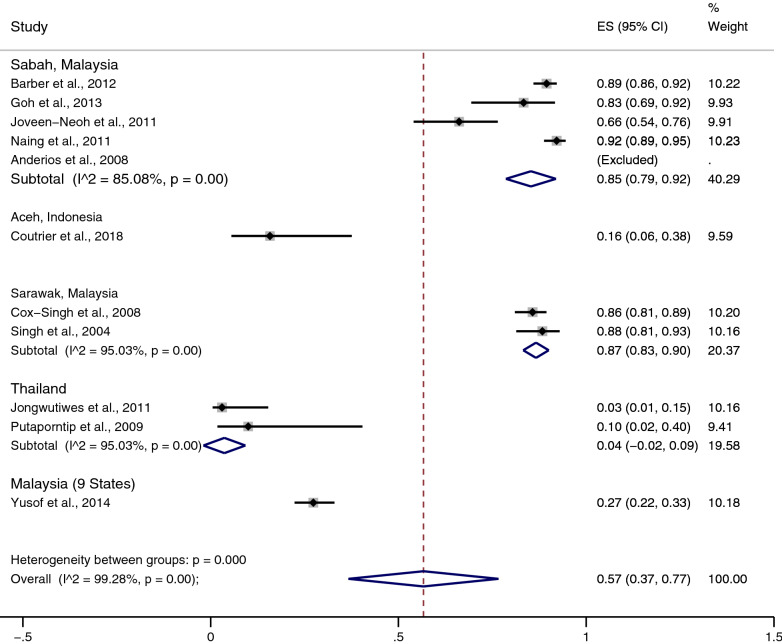
Pooled prevalence of the misidentification of *P. knowlesi* as *P. malariae*. The pooled prevalence of discordance of the misidentification of *P. knowlesi* as *P. malariae* was estimated by a random effects model using the numerator in the prevalence calculation as the number of discordances, and the denominator as the number of PCR-positive malaria cases. *ES* estimated proportion, *CI* confidence interval, *random* random effects model

The total number of instances in which *P. knowlesi* was misidentified as *P. malariae* by microscopy was 1170. Based on the 11 included studies, the pooled prevalence of the misidentification of *P. knowlesi* as *P. malariae* by microscopy was 57% (37–77%, I^2^: 99.3%) (Fig. 3). The highest rate of misidentification of *P. knowlesi* as *P. malariae* was demonstrated in the study by Anderios et al*.* (25/25, 100%) [13], while the lowest rate was by Jongwutiwes et al. (1/33, 3.03%) [24].

### Subgroup analysis of the misidentification of *P. knowlesi* as *P. malariae*

Subgroup analysis of the study sites was performed to demonstrate the differences in the geographical distribution of the misidentification (Fig. 3). The results of the subgroup analysis demonstrated that the pooled prevalence of the misidentification was highest in Sawarak, Malaysia (87%, 95% CI 83–90%, I^2^: 95%, 2 studies), and Sabah, Malaysia (85%, 95% CI 75–92%, I^2^: 85.1%, 5 studies). The study by Yusof et al*.* [16] demonstrated 27% (95% CI 22–33%) misidentification in nine states of Malaysia. A low prevalence of the misidentification of *P. knowlesi* as *P. malariae* was demonstrated in Thailand (4%, 95% CI − 2 to 9%, I^2^: 95%, 2 studies) and Aceh, Indonesia (16%, 95% CI 6–38%).

### Publication bias

The funnel plot could not be generated because few studies were included in the present study. Egger’s test demonstrated that no small-study effect was found (p: 0.126, coefficient: − 11.6, standard error: 6.79), indicating no publication bias among the included studies.

## Discussion

Although *P. knowlesi* is well documented in Malaysia, the pooled quantification of the misidentification of this species as *P. malariae* has not been previously described. In this study, such misidentification was quantified using 11 studies [3, 13–19, 23, 24, 27], and it was found that the rate of this misidentification from 2000 to 2015 was 57%, with high heterogeneity among the included studies. Subgroup analysis of the study sites demonstrated a large difference in the misidentification rates. The highest prevalence of misidentification was demonstrated in two states of Malaysian Borneo, Sabah and Sawarak, where *P. knowlesi* was endemic in the last decade. In these areas, the number of *P. knowlesi* cases was not available until PCR testing was used to identify it in 2004 [3]. Molecular detection assay, nested PCR assay and real-time PCR test have been described for *P. knowlesi* targeting 18S rRNA gene targets [3, 61], with sensitivity of between 1 and 6 parasites/µl of blood [62]. From the time that nested PCR was implemented for diagnosis, the number of cases of *P. knowlesi* significantly increased, while a small number of *P. malariae* cases were still observed by PCR in Sabah during 2008–2011 [14, 15, 18] and in Sawarak during 2001–2006 [19]. This indicated that the highest number of *P. malariae* cases identified by microscopy in the last decade was caused by the emergence of *P. knowlesi* malaria, as these two species are morphologically similar and difficult to distinguish from each other using microscopy. While the highest prevalence of the misidentification of *P. knowlesi* as *P. malariae* occurred in Sabah and Sawarak, microscopically misdiagnosed cases of *P. malariae* were not found in other parts of Malaysia, such as Pahang and Kelantan [16]. This explained why the prevalence of the misidentification of *P. knowlesi* as *P. malariae* in the study by Yusof et al*.* [16] in nine states of Malaysia was lower than in studies conducted in Sabah and Sawarak [3, 13–15, 17–19].

In areas in which *P. knowlesi* was endemic, it was also frequently misidentified as *P. falciparum* or *P. vivax* malaria by microscopy [10]. The similarity of *P. knowlesi* and *P. falciparum* occurs at the stage of young rings of both species, which contain double chromatin dots, multiple-infected erythrocytes, and applique forms [63]; while the similarity of *P. knowlesi* and *P. malariae* occurs in the trophozoite, schizont, and gametocyte stages [63]. The recent decrease in diagnostic discrepancies by microscopy was due to the increased awareness and recognition among microscopists of *P. knowlesi* infections in endemic areas. Moreover, *P. malariae* is less endemic in Southeast Asia, where the presence malarial parasites with morphology similar to that of *P. malariae* coupled with high parasitaemia has been reported as *P. knowlesi* infection by default [16]. Moreover, the World Health Organization (WHO) recommends that all *P. malariae*-positive diagnoses by microscopy in *P. knowlesi* endemic areas be reported as *P. malariae*/*P. knowlesi* [64].

In areas where *P. knowlesi* is not endemic, such as Thailand and Indonesia, a low prevalence of the misidentification of *P. knowlesi* as *P. malariae* by microscopy was observed. Only one case of *P. knowlesi* misidentified as *P. malariae* from 33 confirmed cases of *P. knowlesi* was recorded by Jongwutiwes et al*.* during 2008–2009 [24], and only one such case among ten confirmed cases of *P. knowlesi* was recorded by Putaporntip et al*.* during 2006–2007 [23]. There was also a low prevalence of such misidentification by microscopy during 2014–2015 in Aceh, Indonesia [27], as only three cases of *P. knowlesi* were misdiagnosed from 19 confirmed cases of *P. knowlesi* as recorded by Coutrier et al*.* [27]. In addition, *P. knowlesi* was also misidentified as *P. falciparum* and *P. vivax*, as reported by studies in both Thailand and Indonesia [24, 27]. This indicated that microscopists were unable to recognize *P. knowlesi* because its ring forms were similar to those of *P. falciparum,* or sometimes its growing trophozoites were similar to those of *P. vivax.* Misidentification, such as the misdiagnosis of *P. falciparum* as *P. knowlesi*, might cause the administration of chloroquine, and the resistance of *P. falciparum* to chloroquine can increase the likelihood of patient mortality. Further, the misidentification of severe *P. knowlesi* as *P. vivax* malaria may lead to treatment failure, such as missed parenteral treatments as per national guidelines, which have been reported to be associated with fatal outcomes [56]. In addition to the misidentification of *P. knowlesi* mono-infection, mixed-infections of *P. knowlesi* combined with other *Plasmodium* species were also recorded in Thailand and Indonesia, such as mixed-infections with *P. falciparum* or mixed-infections with *P. vivax* malaria, which microscopists reported as *P. falciparum* or *P. vivax* mono-infections [23, 24, 27]. Severe complications due to *P. knowlesi* malaria in those co-infected patients in non-endemic countries such as Thailand and Indonesia were less likely since low parasite density of *P. knowlesi* was observed [23, 24], and *P. knowlesi* was responsive to chloroquine treatment in cases of mixed infections with *P. vivax* malaria. In addition, severe adverse events from unnecessary primaquine treatments were not experienced among co-infected patients [27].

The present study had some limitations. First, high heterogeneity among the included studies was observed, although subgroup analysis was performed; therefore, the results of the pooled prevalence needed to be interpreted carefully. Second, a low number of included studies were used for pooled analysis; therefore, the pooled prevalence might not have been precisely estimated. Third, studies reporting on *P. knowlesi*/*P. malariae* as determined by microscopy were not included in the present study since the number of misidentifications could not be estimated.

## Conclusion

Although the WHO recommends that all *P. malariae*-positive diagnoses made by microscopy in *P. knowlesi* endemic areas be reported as *P. malariae*/*P. knowlesi*, the possibility of the misidentification of *P. knowlesi* as *P. malariae* by microscopists is a diagnostic challenge in both endemic and non-endemic countries. Assuming the low incidence of *P. malariae* in Malaysia and Southeast Asia, cases of symptomatic malaria with hyperparasitaemia and parasite morphology resembling that of *P. malariae* should be diagnosed as *P. knowlesi*/*P. malariae* by microscopy, so that severe complications among patients infected by *P. knowlesi* can be reduced.

## Supplementary Information

**Additional file 1: Checklist S1.** PRISMA Checklist S1.

**Additional file 2.** Methodological quality summary.

## Data Availability

All data related to the present study are available.
